# Treatment of a stage III rima glottidis patient with the oncolytic virus Rigvir: A retrospective case report

**DOI:** 10.1097/MD.0000000000017883

**Published:** 2019-11-11

**Authors:** Guna Proboka, Agnija Rasa, Evija Olmane, Sergejs Isajevs, Andra Tilgase, Pēteris Alberts

**Affiliations:** a Latvian Oncology Centre, Riga Eastern Clinical University Hospital; b Rigvir; c Department of Radiology, Pauls Stradiņš Clinical University Hospital; d Department of Pathology, Riga Eastern Clinical University Hospital; e Department of Pathology, Faculty of Medicine, University of Latvia, Riga, Latvia.

**Keywords:** ECHO-7 virus, glottis, laryngeal cancer, oncolytic virus, Rigvir

## Abstract

**Rationale:**

Of all the parts of the larynx, the glottis has the highest frequency of cancer. With disease progression, the vocal cord movement is affected and for advanced stages its anatomical and functional preservation is rarely achievable, if at all.

**Patient concerns:**

Here we describe a 72-year-old patient who presented with hoarseness for a year and was only able to whisper.

**Diagnosis:**

A computed tomography (CT) scan of the vocal cords (without contrast) showed higher density tissue. Histological examination disclosed a well-differentiated verrucous squamous cell carcinoma of the glottis.

**Interventions:**

The patient was treated with the oncolytic ECHO-7 virus Rigvir without any of the standard treatments.

**Outcomes:**

As shown by CT scans, the patient has been stabilized, and the laryngeal functions are preserved with the virotherapy still ongoing. The patient was diagnosed over 4.2 years ago.

**Lessons:**

Considering the present patient being treated with Rigvir without any standard treatment, the results suggest that Rigvir therapy could be a possible treatment for glottic cancer.

## 1 Introduction

Laryngeal cancer constitutes about a quarter of all head and neck cancer cases.^[1]^ Smoking and alcohol are considered to be the main risk factors.^[1,2]^ At the time of diagnosis, about 60% of patients have an advanced disease. The 5-year survival rate has decreased during the last 4 decades, indicating that improvements in the disease management are necessary.^[3]^

Standard treatment depends on the stage of the cancer.^[1]^ Historically the primary treatment has been surgery but with time preservation of the larynx and its’ functions have become a matter of importance.^[4]^ Usually, only early-stage patients receive a larynx-preserving treatment approach involving either surgery or radiotherapy, which is available for patients with a small primary tumor. For stage III and IV patients the conventional treatment is a combination of surgery, chemotherapy, and radiotherapy.^[1]^ There are difficulties to maintain a functional larynx even if the larynx itself is anatomically preserved. There is a series of complications, the most often observed after chemotherapy, such as decline in the swallowing function, mucositis, and dysphagia.^[5]^ Many patients are reluctant to perform surgery because of the risk to lose the voice.^[3]^

The larynx is anatomically being divided into 3 sites, the supraglottis, glottis, and subglottis.^[2]^ The glottis is recognized as the most frequent site to develop cancer. Hoarseness, stridor, sore throat, persistent cough, or a neck mass are the most commonly observed symptoms.^[1]^ At stage III, glottic cancer starts to limit the movement of the vocal cord.^[6]^

The aim of this report is to show the effect of Rigvir treatment in a stage III glottis cancer patient that has not received any other treatment. Rigvir is an oncolytic, nonpathogenic and genetically unmodified ECHO-7 virus selected and adapted for melanoma.^[7]^ It has been registered for the treatment of cutaneous melanoma^[8]^ but the present patient was treated off-label.

## 2 Case report

A man born in 1947 was diagnosed with a laryngeal vestibule carcinoma of the rima glottidis stage III (T_3_N_0_M_0_) on July 1, 2015. Presently he maintains a job on a farm as a driver. The patient has been reported to have had scarlet fever in childhood, frequent tonsillitis in adolescence, septoplasty around 1980 and coronary artery bypass surgery in 2011. He is receiving treatment for thrombocytopenia since 2000. The patient has vibration disease because of his previous occupation as a woodworker and has had allergic reactions to diphtheria vaccine and other vaccines. For that reason, he is not vaccinated against any diseases. The patient is a smoker from the age of 18. Previously he smoked 1 pack per day, presently only 3 to 4 cigarettes per day; he has practically never consumed alcohol. The patient has a 70°C sauna every week. There is a family history of cancer; the patients’ brother was diagnosed with esophageal cancer at the age of 56.

**Figure 1:**
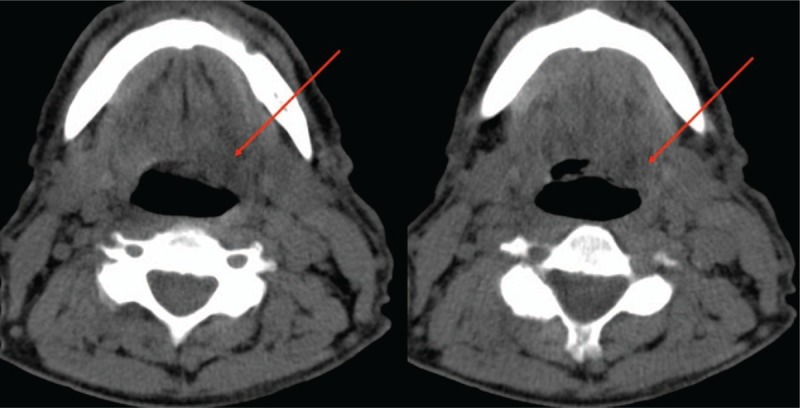
CT (without contrast) scan on 17 December 2014 of the vocal cords shows tissue of higher density (arrows). CT = computed tomography.

The patient had had hoarseness for a year and was only able to whisper. Due to this impediment, the patient visited an otolaryngologist in December 2014. After the examination he was sent to an oncologist for further diagnosis. A computed tomography (CT) scan of the vocal cords (without contrast) was made on December 17, 2014. Higher density tissue on the left side was found (Fig. 1). The examination by the oncologist occurred only on June 15, 2015; biopsy from the left larynx was taken on July 1, 2015.

**Figure 2:**
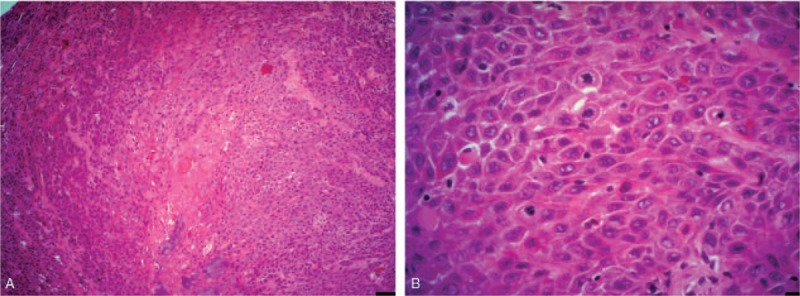
Representative photomicrographs of well-differentiated verrucous squamous cell carcinoma, hematoxylin-eosin staining. (A) Magnification ×200, scale bar 50 μm. (B) Pathological mitosis, magnification ×400, scale bar 25 μm.

Histopathological examination showed that the tumor is composed of squamous epithelial cells with an abundant pink cytoplasm, mild to moderate atypia, well-developed keratinization, with mild stromal desmoplasia, and moderate peritumoral inflammatory cell infiltration. The mitotic count was up to 5 per mm^2^. The diagnosis of well-differentiated verrucous squamous cell carcinoma was made (Fig. 2).

Taking into consideration the tumor distribution, the patient was offered to undergo laryngectomy and radical radiotherapy. The patient refused both treatments. After searching for other cancer treatment options, the patient found out about Rigvir virotherapy and that it was available in Latvia for treatment of melanoma. Therefore, he chose Rigvir virotherapy.

Rigvir therapy was started on July 15, 2015 with 3 intramuscular administrations for 3 consecutive days. Subsequently, the administrations were once every 3 weeks. From November 2015, the administrations were 1 administration per month, from December 2016 1 administration every 6 weeks, from May 2017 1 administration every 2 months, and from December 2017 1 administration every 2 weeks; the virotherapy is still ongoing. The patient has not received any other treatment.

Serum clinical chemistry parameters (13 times from March 2015 to December 2017) were recorded and graded accordingly to National Cancer Institute Common Terminology Criteria for Adverse Events. Values above grade 2 were not observed during Rigvir therapy. Decreased thrombocyte levels were detected (grade 1 and 2). This disorder is not related to the treatment since the patient had thrombocytopenia even before the start of Rigvir therapy.

**Figure 3:**
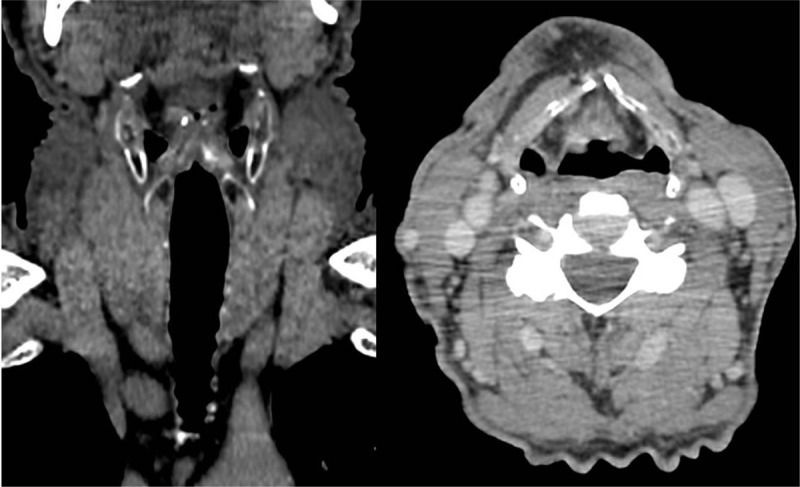
Contrast-enhanced CT scan on February 8, 2018 shows no signs of dissemination or local recurrence. CT = computed tomography.

A follow-up contrast-enhanced CT scan of the neck, thorax, and abdomen was made on 8 February 2018. No signs of a local recurrence or dissemination were detected (Fig. 3). A laryngoscopy (March 27, 2018) was also made. Tissue growth or thickening was not visible. The hoarseness has decreased. His voice is still altered likely due to long-term smoking.

## 3 Discussion

To our knowledge, this is the first case of oncolytic virotherapy as a monotherapy for laryngeal cancer. For laryngeal cancer the treatment options are limited, involving only surgery as the main treatment and chemoradiotherapy, not guaranteeing the functionality of the larynx. And in many cases cancer cells can become resistant to radiotherapy and chemotherapy, which accounts for high recurrence rates.^[9]^ Overexpression of the epidermal growth factor receptor (EGFR) has been acknowledged as a valid factor to cause radioresistance. This overexpression is noticeable in majority of head and neck squamous cell carcinoma patients and is said to correlate with radioresistance observed in both mouse adenocarcinoma models and cancer patients.^[10,11]^ Alongside EGFR, EGFR variant III (EGFRvIII), a mutant form of EGFR, can also be expressed. EGFRvIII is known for its’ resistance to targeted treatment.^[12,13]^

Oncolytic viruses have been viewed as a possible treatment for head and neck cancers, the most frequently assessed being the head and neck squamous cell carcinoma (HNSCC). HNSCC patients mainly have decreased clusters of differentiation (CD)3^+^, CD4^+^, and CD8^+^ T cell levels, which worsens the prognosis.^[14]^ Oncolytic viruses activate the immune response against the tumor by entering and infecting the tumor cells and releasing tumor-associated antigens, pathogen-associated molecular patterns, and cellular danger-associated molecular patterns. As a result, antigen-specific CD4^+^ and CD8^+^ T cell responses are activated.^[15]^ Numerous viruses have been involved in clinical trials to investigate the efficacy in head and neck cancers. Talimogene laherparepvec (T-VEC) has demonstrated encouraging results. In a phase I and phase II dose-finding study in locally advanced HNSCC patients were offered chemoradiation therapy and T-VEC, showing disease-specific survival of 82% and relapse-free survival of 76%.^[16]^ A study evaluating the efficiency of herpes simplex virus NV1020 in 5 HNSCC lines showed sensitivity towards NV1020. Nearly complete regression was observed in 2 cell lines and a significant partial regression in 1 cell line.^[17]^

Smoking is considered as the most important risk factor and can be associated with many health disorders. It can even affect the disease-specific survival time and the efficacy of treatment.^[18]^ Considering that the present patient is a smoker and did not discontinue smoking even after the diagnosis and during the treatment, the disease outcome could be considered as noteworthy. As pointed out by a clinical review, continuous smoking after cancer treatment worsens the prognosis by 21% to 35% compared to patients that recessed.^[19]^

Regarding the possible cause of the cancer, wood dust may have had a role in the development of the cancer because of the patients’ previous occupation as a woodworker. Wood dust is considered to be carcinogenic to humans. In a case-control study the laryngeal cancer risk from wood dust exposure was assessed. An elevated, dose-dependent risk of malignancy was found.^[20]^

The 5-year overall survival for T3 patients varies depending on the selected treatment. Analysis of laryngeal cancer patients showed when treated with total laryngectomy and postoperative radiation- or chemotherapy, the 5-year overall survival was 70%, with radiation therapy alone 18%, and with chemoradiotherapy-radiotherapy 52%.^[21]^ According to the American Joint Committee on Cancer Cancer staging manual, the 3- and 5-year relative overall survival for squamous cell carcinoma of the glottis stage III are 64% and 55.7% when treated with standard of care in the USA.^[22]^ The present patient was diagnosed over 4.2 years ago and has only been treated with Rigvir without surgery, chemotherapy or radiotherapy, accomplishing stabilization with maintained function of the vocal cords and without side effects. This case report suggests that virotherapy with Rigvir could be a possible treatment. More research in this field is necessary.

## 4 Declarations

### 4.1 Patient consent statement

Written consent for publication of anonymized data has been obtained from the patient.

### 4.2 Ethical approval

Off-label has been defined as “all uses of a marketed drug not detailed in the summary of product characteristics including therapeutic indication, use in age-subsets, appropriate strength (dosage), pharmaceutical form and route of administration,”^[23]^ and a “different indication in term of medical condition than the^[1]^ described in the authorised product information; a different group of patients than the^[1]^ described in the authorised product information; a different route or method of administration than the^[1]^ described in the authorised product information; a different posology than the^[1]^ described in the authorised product information.”^[24]^ The off-label use in oncology for adults has been reported to be in the range 10% to 76%, depending on cancer.^[25]^ Consequently, in the case reported here, the off-label use is not part of a prospective study but part of daily practice and as such does not require the approval of an ethical committee.

## Author contributions

**Conceptualization:** Guna Proboka.

**Data curation:** Guna Proboka, Agnija Rasa, Andra Tilgase, Pēteris Alberts.

**Formal analysis:** Agnija Rasa, Evija Olmane, Sergejs Isajevs, Andra Tilgase, Pēteris Alberts.

**Writing – original draft:** Agnija Rasa, Andra Tilgase, Pēteris Alberts.

**Writing – review and editing:** Guna Proboka, Agnija Rasa, Evija Olmane, Sergejs Isajevs, Andra Tilgase, Pēteris Alberts.

Agnija Rasa orcid: 0000-0001-6561-9371.
