# Treatment of a stage III rima glottidis patient with the oncolytic virus Rigvir: A retrospective case report: Erratum

**DOI:** 10.1097/MD.0000000000018401

**Published:** 2019-12-10

In the article, “Treatment of a stage III rima glottidis patient with the oncolytic virus Rigvir: A retrospective case report”,^[1]^ which appears in Volume 98, Issue 45 of *Medicine*, the second quotation in 4.2. Ethical approval is written incorrectly and should be “different indication in term of medical condition than the one described in the authorised product information; a different group of patients than the one described in the authorised product information; a different route or method of administration than the one described in the authorised product information; a different posology than the one described in the authorized product information.”
